# RNA profile of control and nicotine treated Murine Leydig single cell represented in TPM data

**DOI:** 10.1016/j.dib.2019.103825

**Published:** 2019-03-27

**Authors:** Jiajie Wu, Wangjie Xu, Dong Zhang, Jingbo Dai, Yong Cao, Yilin Xie, Lianyun Wang, Zhiguang Qiao, Zhongdong Qiao

**Affiliations:** a School of Life Science and Biotechnology, Shanghai Jiao Tong University, Shanghai, 200240, PR China; b Shanghai Ninth People's Hospital, School of Medicine, Shanghai Jiao Tong University, Shanghai 200011, China; c Feinberg School of Medicine, Northwestern University, Chicago, IL, 60611, United States

## Abstract

Data provided in this article is RNA profile represented in RPKM and RPKM based TPM value for the research article titled Nicotine inhibits Murine Leydig cell differentiation and maturation via regulating Hedgehog signal pathway Jiajie et al., 2019. Nicotine treatment changes the RNA profile of Murine Leydig cells. RNA of 12 control group Leydig cells and 12 nicotine treated Leydig cells are sequenced and the data of 29943 genes are achieved. The information of the gene symbol, gene description, gene type, position and transcript length are provided.

**Table:** Specifications table

Subject area	*Biology*
More specific subject area	*Molecular Biology*
Type of data	*Table*
How data was acquired	*Single cell RNA-seq via C1 single cell auto prep system from Fluidigm and Illumina system*
Data format	*Raw*
Experimental factors	*Control and nicotine treated groups*
Experimental features	*Get the RNA profile of control and nicotine treated group Leydig cell.*
Data source location	*School of Life Science and Biotechnology, Shanghai Jiao Tong University, Shanghai, China*
Data accessibility	*Data is attached with this article*
Related research article	*Jiajie Wu, Wangjie Xu, Dong Zhang, Jingbo Dai, Yong Cao, Yilin Xie, Lianyun Wang, Zhiguang Qiao, Zhongdong Qiao, Biochemical & Biophysical Research Communications, 2019 Feb 26;510(1):1–7.*https://doi.org/10.1016/j.bbrc.2018.11.107*. Epub 2019 Jan 23.*; [1]*.*

**Table:** 

**Value of the data** • This data provides the full view of the RNA profile of Leydig cell, which can be compared with some other cell types in male Murine, so that the specific characteristics of the Leydig cells can be explored. • The data consist of RPKM and TPM value of 24 single Leydig cells, which can be helpful in understanding differences in specific single cells and the general character as well. • The comparison between the control and nicotine treated groups contribute in understanding the influence of nicotine on Leydig cells via many aspects.

## 1 Data

The data shows the RPKM and TPM value of 29943 genes of 12 control group Leydig cells and 12 nicotine treated Leydig cells as the attached Table all_SC_SCT_ANNOTATION_TPM. It provides gene ID, NCBI enterz, gene symbol, gene description, gene type, gene position on chromosome and transcript length. SCT refers to nicotine treated group and SC refers to control group. And SCT/SC_TPM or SCT/SC_RPKM are the TPM and RPKM value of each gene.

## 2 Experimental design, materials, and methods

The TPM value of 24 single Leydig cells were acquired separately from control group and nicotine treated group C57BL/6J mice. All the mice were 6-week-old which were sexually matured [2]. The treating of nicotine lasted for 5 weeks which was the complete spermatogenic cycle of a mouse [2]. The treating amount was 0.2mg/100g/day nicotine which was the mimic amount as the heavy smokers [3].

After collecting the Leydig cells of mice via density gradient centrifugation, the single cells were prepared with the C1 single cell auto prep system of Fluidigm. The process was conducted according to the instruction of Fluidigm C1 Single-Cell Auto Prep Array IFC.

The RNA of the single cells were extracted and reverse transcribed into cDNA with Fluidigm system. And the following sequencing was carried out by TD202-TruePrep Index Kit V2 for Illumina. The converting from reads to RMKP follows the following equation:

$$\text{RPKM}=\frac{\text{total}\;\;\text{exon}\;\;\text{reads}}{\text{mapped}\;\;\text{reads}\;\text{(millions)}\;\text{*}\;\text{exon}\;\;\text{length}\;\text{(KB)}}$$

To realize normalization of the sequencing result, TPM based on RPKM is adopted to show the expression level of the transcripts.

$$\text{TPM}=\frac{\text{RPKM*}1000000}{\text{total}\;\text{RPKM}}$$

## References

[1] Wu Jiajie, Xu Wangjie, Zhang Dong (2019 Feb 26). Nicotine inhibits Murine Leydig cell differentiation and maturation via regulating Hedgehog signal pathway. Biochem. Biophys. Res. Commun..

[2] Aitken R.J., Findlay J.K., Hutt K.J. (2011). Apoptosis in the germ line. Reprod..

[3] Matta S.G., Balfour D.J., Benowitz N.L. (2007). Guidelines on nicotine dose selection for in vivo research. Psychopharmacol..

